# Role of the β-Catenin/REG Iα Axis in the Proliferation of Sessile Serrated Adenoma/Polyps Associated with *Fusobacterium nucleatum*

**DOI:** 10.3390/pathogens10040434

**Published:** 2021-04-06

**Authors:** Heihachiro Nishimura, Hirokazu Fukui, Xuan Wang, Nobuhiko Ebisutani, Takashi Nakanishi, Toshihiko Tomita, Tadayuki Oshima, Seiichi Hirota, Hiroto Miwa

**Affiliations:** 1 Division of Gastroenterology and Hepatology, Department of Internal Medicine, Hyogo College of Medicine 1-1, Mukogawa, Nishinomiya 663-8501, Japan; he-nishimura@hyo-med.ac.jp (H.N.); xwang31@tmu.edu.cn (X.W.); no-ebisutani@hyo-med.ac.jp (N.E.); ta-nakanishi@hyo-med.ac.jp (T.N.); tomita@hyo-med.ac.jp (T.T.); t-oshima@hyo-med.ac.jp (T.O.); miwahgi@hyo-med.ac.jp (H.M.); 2 Department of Surgical Pathology, Hyogo College of Medicine 1-1, Mukogawa, Nishinomiya 663-8501, Japan; hiros@hyo-med.ac.jp

**Keywords:** *Fusobacterium nucleatum*, sessile serrated adenoma, polyp, β-catenin, REG, Ki67, proliferation

## Abstract

Although sessile serrated adenoma/polyps (SSA/Ps) may arise through a pathway different from the traditional adenoma–carcinoma sequence, details of SSA/P tumorigenesis still remain unclear. *Fusobacterium nucleatum* (*Fn*) is frequently detected in colorectal cancer (CRC) tissues and may play a pivotal role in colorectal carcinogenesis. Here, we investigated the relationship between *Fn* and the β-catenin/REG Iα axis in SSA/Ps and their involvement in the proliferation of these lesions. *Fn* was detected in SSA/Ps by fluorescence in situ hybridization using a *Fn*-targeted probe, and expression of β-catenin, REG Iα and Ki67 was examined using immunohistochemistry. Sixteen of 30 SSA/P lesions (53.3%) were positive for *Fn*. Eighteen SSA/P lesions (60%) showed β-catenin immunoreactivity in the tumor cell nuclei. A significant majority of *Fn*-positive lesions showed nuclear expression of β-catenin (87.5%) and higher REG Iα scores and Ki67 labeling indices relative to *Fn*-negative lesions. The SSA/P lesions expressing β-catenin in nuclei had significantly higher REG Iα scores and Ki67 labeling indices than those expressing β-catenin on cytomembranes. The REG Iα score was positively correlated with the Ki67 labeling index in SSA/P lesions. The treatment with Wnt agonist SKL2001 promoted nuclear β-catenin translocation and enhanced *REG Ia* expression in Caco2 cells. *Fn* may play a role in the proliferation of SSA/P lesions through promotion of β-catenin nuclear translocation and REG Iα expression.

## 1. Introduction

Although it is widely accepted that colorectal cancers (CRCs) arise from adenomas [1], the pathway responsible has recently been shown to be far from simple [2]. Among colorectal adenomas, sessile serrated adenoma/polyps (SSA/Ps) are classified as a subgroup exhibiting a specific saw-toothed colonic crypt morphology and genetic alterations such as *BRAF* mutation and microsatellite instability [3,4]. Furthermore, SSA/Ps lesions show high-labeling index for Ki67 expression [5], suggesting that those lesions have high ability in cell proliferation. These morphologic and genetic alterations are quite different from those in conventional adenomas, from which CRCs arise through accumulation of *APC*, *KRAS* and *p53* mutations in multiple steps [1]. Thus, the molecular mechanism whereby CRCs arise and progress from adenomas is not fully clear. In addition to genetic alterations, the role of the gut microbiome in colorectal carcinogenesis has been highlighted; microbiome imbalance (dysbiosis) is closely associated with the development and progression of CRCs through promotion of chronic inflammatory conditions and production of carcinogenic metabolites [6]. Among various candidate pathogenic bacteria, *Fusobacterium nucleatum* (*Fn*) has been gathering the most attention, since numerous studies have reported that a higher abundance of *Fn* is associated with a more advanced stage, a higher risk of recurrence, and shorter survival in patients with CRC [7,8,9]. However, it is still debatable whether *Fn* directly plays a role in the pathogenesis of CRC patients although *Fn* may promote colorectal carcinogenesis by activating β-catenin signaling in vitro experiments [10,11].

The regenerating gene (REG) was first isolated from rat regenerating pancreatic islets [12], and since then, many REG-related genes have been isolated, currently constituting a family with multiple members (types I-IV) [13,14]. We and others have reported that REG family proteins play some roles in not only inflammatory [15,16,17,18] but also neoplastic [19,20,21] diseases of the gastrointestinal (GI) tract. In fact, REG Iα protein, as well as other REG family proteins [22,23,24], are suggested to exert a cell proliferative and/or anti-apoptotic effect in inflamed and neoplastic lesions in the GI tract [25,26,27]. In relation to the up-regulation of REG Iα expression in GI neoplasia, we have previously clarified that STAT3-associated cytokines play a pivotal role in REG Iα overexpression in gastric cancer cells [28]. However, the mechanism by which REG Iα overexpression is regulated in neoplastic lesions remains unclear. On the other hand, it is well known that β-catenin signaling is one of the major pathways of carcinogenesis in colorectal tumors, including SSA/Ps [29,30]. In this regard, it is interesting to speculate that β-catenin mutation and aberrant β-catenin expression may be linked to REG Iα overexpression in hepatocellular carcinoma, colorectal tumors, or salivary tumors [31,32]. In the present study, therefore, we focused on the relationship between *Fn* abundance and the β-catenin/REG Iα axis and investigated its significance for the proliferative ability of SSA/P lesions.

## 2. Results

### 2.1. Detection of Fusobacterium Nucleatum in SSA/P Lesions

**Figure 1:**
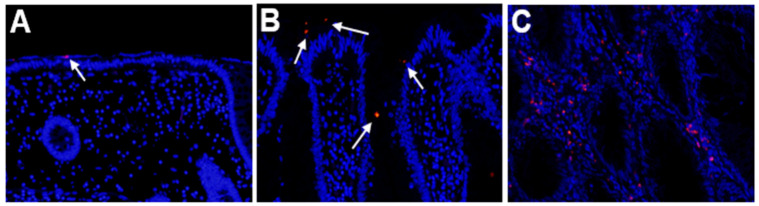
Detection of *Fusobacterium nucleatum* (*Fn*) by fluorescence in situ hybridization. (**A**) Non-neoplastic colonic mucosa adjacent to a sessile serrated adenoma/polyp (SSA/P) lesion. (**B**,**C**) SSA/P lesions. The signals (red dots) for *Fn* are evident at the surface (**B**) or lamina propria (**C**) of the SSA/P lesions but are hardly evident in the non-neoplastic mucosa. Signals are indicated by white arrows.

The FISH signal for *Fn* was detected not only on the luminal surface but also the lamina propria of SSA/P lesions (Figure 1). We evaluated the density of the signal population (magnification ×200) in both SSA/P lesions and in the adjacent non-neoplastic regions. As the density in the non-neoplastic regions was 0.81 ± 0.15 (maximum 2.50), we considered any SSA/P lesion to be positive for *Fn* if the signal density was more than 3.0. Based on this definition, 16 of 30 SSA/P lesions (53.3%) were positive for *Fn* in the samples examined. None of the determined parameters—age, gender, tumor location, or endoscopic morphology—showed a significant relationship to *Fn* positivity.

### 2.2. Expression of REG Iα and β-Catenin in SSA/P Lesions

**Figure 2:**
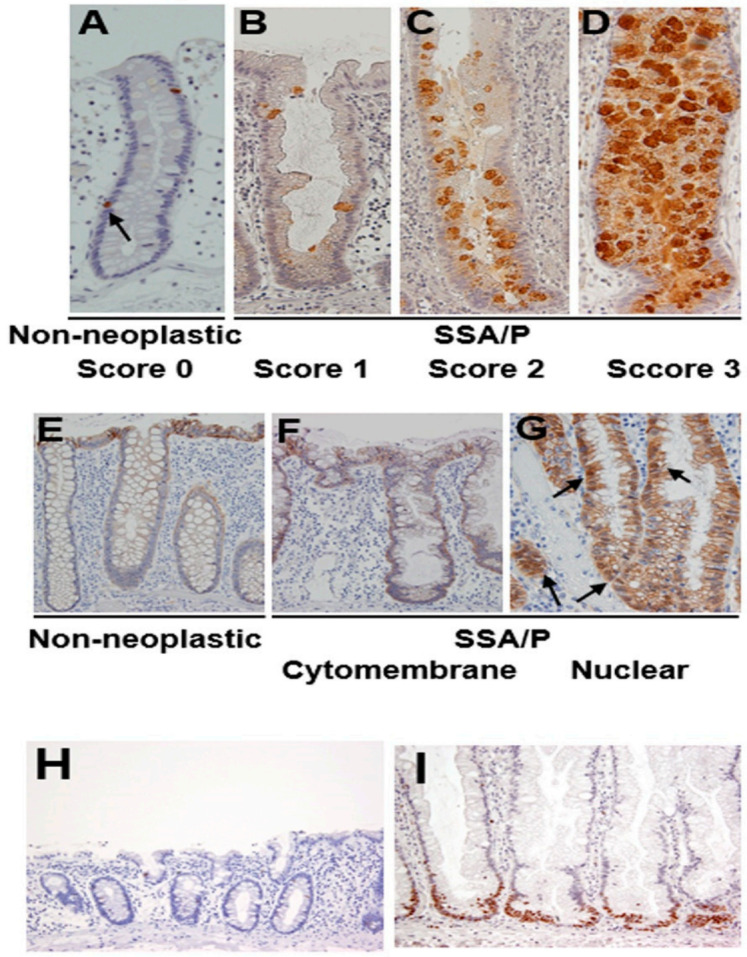
Expression of REG Iα, β-catenin and Ki67 in SSA/P and its adjacent non-neoplastic mucosa in the colon. (**A**–**D**) Grading of REG Iα expression in SSA/P lesions and non-neoplastic colonic mucosa. (**E**–**G**) β-catenin expression pattern in SSA/P lesions and non-neoplastic colonic mucosa. Arrows indicate nuclear expression of β-catenin. (**H**,**I**) Expression of Ki67 in SSA/P lesions and non-neoplastic colonic mucosa.

REG Iα was expressed in a few epithelial cells in non-neoplastic crypts in the colonic mucosa (Figure 2A). In SSA/P lesions, REG Iα was expressed mainly in goblet cells, and we scored its expression as described in materials and methods. All of the SSA/P lesions showed apparently higher scores for REG Iα expression than their adjacent non-neoplastic areas (Figure 2A–D). The REG Iα expression score was significantly higher in SSA/Ps on the right side of the colon (1.71 ± 0.11, *p* < 0.05) than in those on the left side of the colon (0.90 ± 0.31). None of the remaining parameters had any significant relationship to the REG Iα expression score.

Immunoreactivity for β-catenin was detected in the cytomembranes of non-neoplastic epithelial cells in the colonic mucosa (Figure 2E). In SSA/P lesions, β-catenin immunoreactivity was detected in not only cytomembranes (Figure 2F) but also the nuclei of serrated epithelial cells (Figure 2G). Twelve of the 30 SSA/P lesions (40%) showed a cytomembrane-type β-catenin staining pattern, whereas 18 of them (60%) showed nuclear-type immunostaining. None of the clinicopathological parameters examined had a significant relationship to the β-catenin expression pattern.

Ki67 was sparsely expressed in the lower portion of non-neoplastic crypts in the colonic mucosa (Figure 2H). On the other hand, Ki67 immunoreactivity was apparently increased, mainly at the bottom of serrated glands (Figure 2I). None of the clinicopathological parameters examined showed a significant relationship to the Ki67 labeling index.

### 2.3. Relationships among REG Iα/β-Catenin Expression, Fusobacterium Nucleatum Abundance, and Proliferative Activity in SSA/P Lesions

We investigated the relationship between *Fn* positivity and REG Iα/β-catenin expression in SSA/P lesions (Table 1). The REG Iα expression score was significantly higher in *Fn*-positive SSA/P lesions. *Fn*-positive SSA/P lesions showed a significant preponderance of nuclear-type β-catenin expression, whereas *Fn*-negative SSA/P lesions tended to show cytomembrane-type expression. In terms of cell proliferative ability, the Ki67 labeling index was significantly higher in *Fn*-positive than in negative SSA/P lesions.

**Figure 3:**
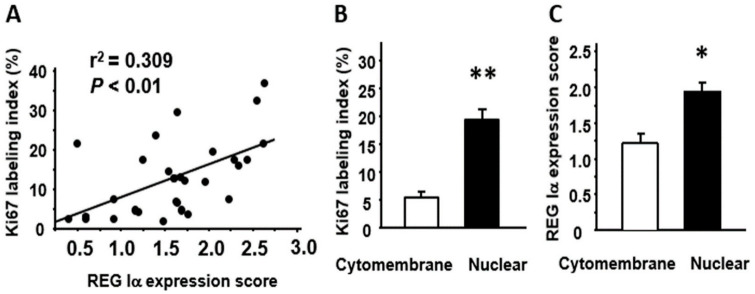
Relationships among REG Iα, β-catenin, and Ki67 expression in SSA/P lesions. (**A**) Correlation between the REG Iα expression score and the Ki67 labeling index in SSA/P lesions. (**B**) Comparison of Ki67 labeling index between SSA/P lesions with cytomembrane-type β-catenin expression and those with nuclear-type expression. (**C**) Comparison of REG Iα expression score between SSA/P lesions with cytomembrane-type β-catenin expression and those with nuclear-type expression. Results are expressed as the mean ± SE. * *p* < 0.005, ** *p* < 0.001 vs. cytomembrane-type group.

We then investigated the correlation between REG Iα/β-catenin expression and the Ki67 labeling index. As shown in Figure 3A, the REG Iα expression score was significantly correlated with the Ki67 labeling index. In addition, SSA/P lesions with nuclear β-catenin expression showed a significantly higher Ki67 labeling index than lesions with cytomembrane expression (Figure 3B). These findings suggested that expression of REG Iα and nuclear β-catenin was linked to the proliferative ability of SSA/P lesions. It was also evident that SSA/P lesions with nuclear β-catenin expression had a significantly higher REG Iα score than lesions with cytomembrane expression (Figure 3C).

### 2.4. Relationship among Histology, Tumor Size, and REG Iα/β-catenin/Ki67 Expression in SSA/P Lesions

Since β-catenin nuclear translocation may occur in SSA/P at the transition to dysplasia [29], we investigated the relationships among pathological features and REG Iα/β-catenin/Ki67 expression in SSA/P lesions. Subsequently, the SSA/Ps with dysplastic change significantly showed nuclear β-catenin expression and, moreover, had a significantly higher score for REG Iα expression (Table 2). In addition, those SSA/Ps tended to show higher Ki67 labeling index compared with ones without. Regarding the size of SSA/P, we found no significant relations to β-catenin (cytomembrane vs. nuclear: 23.4 ± 2.5 vs. 19.5 ± 1.8 mm), REG Iα (r^2^ = 0.02, *p* = 0.45), or Ki67 (r^2^ = 0.03, *p* = 0.88) expression in this study.

### 2.5. Effect of Wnt Signaling on Nuclear β-Catenin Translocation and REG Iα Expression in Caco2 Cells

**Figure 4:**
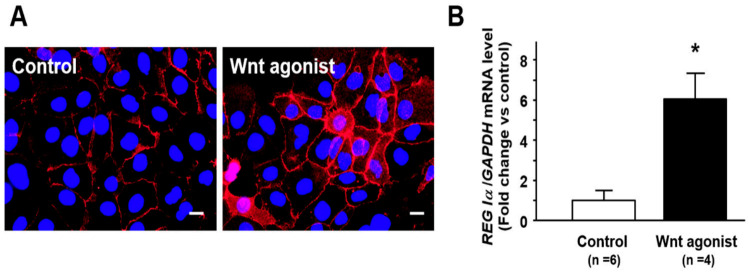
Effect of Wnt agonist SKL2001 on nuclear β-catenin translocation and REG Iα expression in Caco2 cells. (**A**) Representative photograph showing nuclear β-catenin (red) translocation in Caco2 cells treated with Wnt agonist SKL2001. Caco2 cells (5 × 10^4^) were cultured in 12-well plates for 24 h and then treated with Wnt agonist SKL2001 (40 mM) for 24 h. After stimulation, the cells were fixed with methanol for 10 min at room temperature, incubated with primary anti-β-catenin antibody for 1 h, followed by TRITC-labeled secondary anti-rabbit antibodies for 1 h, nuclear counterstained using Antifade Mountant with DAPI (blue) and observed under Olympus BX53 microscope. Bar = 20 mm. (**B**) Change in REG Iα expression in in Caco2 cells treated with Wnt agonist SKL2001. Results are expressed as the mean ±SE. * *p* < 0.05 vs. control group.

When Caco2 cells were stimulated with Wnt agonist SKL2001, the immunoreactivity for β-catenin was detected not only at the cytomembrane but also in the cytoplasm and the nuclei in the cells, suggesting that nuclear β-catenin translocation was promoted in those cells (Figure 4A). Furthermore, the expression of *REG Iα* was significantly enhanced in Caco2 cells by the treatment with Wnt agonist SKL2001 (Figure 4B).

## 3. Discussion

The mechanism of CRC development has been largely studied by focusing on genetic alterations, but recent investigators have begun to recognize the role of the gut microbiome in this respect [6], similarly to the role of *Helicobacter pylori* in gastric cancer development [33]. So far, comprehensive analyses of the gut microbiome have identified several candidate bacteria that may play a role in the development of CRC, and among them, *Fn* has received special attention [7,8,9]. In the present study, we were able to detect the presence of *Fn* in approximately half of the SSA/P lesions we examined, in agreement with a previous report [34]. One limitation in this study was that we were unable to address the mechanism whereby *Fn* affects the carcinogenesis of CRC. However, since SSA/P lesions are known to show high proliferative ability [5], we investigated the relationship between *Fn* positivity and proliferative ability in SSA/P lesions and subsequently clarified that *Fn*-positive lesions had higher proliferative ability than *Fn*-negative lesions. In addition to proliferative ability, *Fn* infection appears to accelerate inflammation and DNA damage in colonic epithelial cells, and those accelerations may be regulated by a specific DNA glycosylase [35]. These findings suggest that not only cell proliferation, but also inflammation-associated DNA damage may be a key to understand the effect of *Fn* infection on the progression of malignant potential in SSA/P lesions.

Of note, β-catenin nuclear translocation is likely to occur in SSA/P lesions at the transition to dysplasia [29,36], being compatible with our obtained data in this study. This finding may suggest that nuclear β-catenin expression may be a marker of high risk of malignant progression in SSA/P lesions. Interestingly, recent studies have clarified that *Fn* is likely to promote CRC growth through the formation of a FadA-E-cadherin-annexin A1-β-catenin complex to activate the nuclear translocation of β-catenin [10,11]. Furthermore, another study has demonstrated that *Fn* may promote the nuclear translocation of β-catenin via a TLR4/P-PAK1 cascade in colorectal cancers [37]. In this respect, it was noteworthy in this study that *Fn*-positive SSA/P lesions showed significant nuclear immunoreactivity for β-catenin. Moreover, we found that SSA/P lesions with a nuclear β-catenin immunostaining pattern had a significantly higher Ki67 labeling index than lesions with cytomembrane immunostaining, indicating that SSA/P lesions with nuclear β-catenin expression had higher proliferative ability. As we demonstrated in this study, β-catenin is normally localized on the cytomembranes of non-neoplastic epithelial cells in the colonic mucosa. However, nuclear translocation of β-catenin is often observed in various tumors, and such translocated β-catenin acts as a transcriptional factor to regulate the expression of its target genes [38,39]. Although we cannot explain exactly why SSA/P lesions with a nuclear β-catenin immunostaining pattern have higher proliferative ability, we speculate that *Fn*-associated β-catenin nuclear translocation may play at least some role in the proliferation of SSA/P lesions. Moreover, it has been known that *APC* mutations and *APC*-related abnormalities are less common in SSA/Ps [40,41]. Conversely, this suggests that *APC*-unrelated β-catenin activation, such as *Fn*-associated ones, may play a pivotal role in progression of SSA/P lesions.

Vigorous mucin production is one characteristic of SSA/P lesions [41,42]. Of note, REG Iα protein is overexpressed in precancerous metaplasia and adenoma that actively express various mucin phenotypes [19,26,43]. Moreover, REG Iα protein functions as a growth and/or anti-apoptotic factor in gastrointestinal tumors [26,27,43], which prompted us to investigate REG Iα expression in SSA/P lesions. In non-neoplastic crypts, REG Iα is expressed in a few endocrine cells with an ovoid or pyramidal morphology, whereas all of the present SSA/P lesions apparently overexpressed REG Iα protein. Interestingly, the REG Iα expression score was positively correlated with the Ki67 labeling index in SSA/P lesions. This suggests that REG Iα expression is associated with the proliferative ability of these lesions, which would be reasonable in view of the known cell growth effect of REG Iα protein. However, one might be concerned whether REG Iα overexpression is specific in SSA/Ps that predominantly arise in the right-side colon. In this regard, a few studies have reported that a proportion of colonic conventional adenomas also overexpress REG Iα protein [44,45]. Therefore, we may have to investigate REG Iα expression in not only SSA/Ps but also conventional adenomas while analyzing the relationship between REG Iα expression and various clinicopathological features.

Finally, we investigated the relationship between β-catenin and REG Iα protein in SSA/P lesions and clarified for the first time that *Fn* positivity was positively correlated with not only nuclear β-catenin expression but also the REG Iα expression score. Moreover, it was noteworthy that SSA/P lesions with nuclear β-catenin expression had a significantly higher REG Iα expression score. These finding suggest that nuclear translocation of β-catenin may be linked to enhancement of REG Iα expression in SSA/P lesions. In this context, previous in vitro studies have shown that β-catenin signaling is responsible for REG Iα expression [46], and that β-catenin mutation may be linked to aberrant REG Iα overexpression in hepatocellular carcinoma [31]. Thus, since both β-catenin and REG Iα are certainly involved in the progression of CRCs, the β-catenin/REG Iα axis may play a pivotal role in the growth of SSA/P lesions. One limitation of the present study was that signaling from *Fn* to the β-catenin/REG Iα axis was not fully investigated. However, we preliminarily showed that nuclear β-catenin translocation by Wnt signaling activation may be closely associated with the enhancement of REG Iα expression in colon cancer cells *in vitro*. Interestingly, recent studies have suggested that Wnt signaling may be a key pathway for *Fn*-associated cell growth in colon cancer [11,47,48]. Thus, since REG Iα is able to function as a growth factor [25,26], we would like to speculate that *Fn*-associated β-catenin-nuclear translocation may play a role in the growth of SSA/P lesions, at least in part, via the growth-promoting effect of REG Iα protein.

In summary, we have demonstrated that nuclear β-catenin expression and REG Iα overexpression are simultaneously evident in *Fn*-positive SSA/P lesions and that these are commonly correlated with the proliferative ability in such lesions. Although we were unable to investigate the pathway involved in signaling from *Fn* to the β-catenin/REG Iα axis, the present findings at least suggest that *Fn* affects the proliferation of SSA/P lesions accompanied by promotion of β-catenin nuclear translocation and REG Iα expression.

## 4. Materials and Methods

### 4.1. Tissue Specimens and Clinicopathological Examination

Thirty patients with SSA/P who underwent endoscopic submucosal dissection at Hyogo College of Medicine Hospital between 2016 and 2019 were enrolled. The SSA/P lesions examined were collected by endoscopic submucosal dissection (n =16), endoscopic mucosal resection (n = 10) or polypectomy (n = 4). The tissue specimens obtained were fixed in 10% buffered formalin and embedded in paraffin, then cut into sections for pathological examination and immunohistochemistry. The characteristics of the patients and their lesions are listed in Table 3. The histological features of dysplastic change were assessed according to the previous descriptions [29,36]. This work was done with the approval of the Ethics Committee of Hyogo College of Medicine.

### 4.2. Immunohistochemistry

Immunohistochemical staining for REG Iα, Ki67 and β-catenin was performed with an Envision Kit (Dako Agilent Technologies, Tokyo, Japan) as described previously [49], using anti-REG Iα antibody (dilution; 1:2000), anti-Ki67 antibody (Dako Agilent Technologies, dilution; 1:50), and anti-β-catenin antibody (Cell Signaling Technology, Danvers, MA, USA; dilution; 1:500). In brief, the rehydrated sections were treated by microwave heating for 20 min in 1×Dako REAL Target Retrieval Solution (Dako Agilent Technologies) and then preincubated with 0.3% H_2_O_2_ in methanol for 20 min at room temperature to quench endogenous peroxidase activity. The sections were then incubated with primary antibodies for 60 min at room temperature, washed in PBS, and incubated with horseradish peroxidase-conjugated secondary antibody for 30 min. The slides were visualized using 3,3′-diaminobenzidine tetrahydrochloride with 0.05% H_2_O_2_ for 3 min and then counterstained with Mayer’s hematoxylin.

### 4.3. Evaluation of Immunostaining

For evaluation of immunohistochemical expression, crypts that were well oriented perpendicularly from the bottom to the surface of the colorectal epithelium were selected. The expression of REG Iα was graded as previously described [50] with minor modification. Thus, it was scored according to the percentage of positive cells in a crypt as follows: score 0, a few cells; score 1, <10%; score 2, 10–50%; score 3, >50%. The Ki67 labeling index was expressed as the percentage of positive cells in a crypt. At least five different visual fields for each SSA/P lesion were observed, and the average REG Iα score and Ki67 labeling index were calculated. β-catenin immunoreactivity was detected in the cytomembranes and nuclei of neoplastic cells in SSA/P lesions. When an SSA/P lesion showed β-catenin immunoreactivity not only in the cytomembranes but also the nucleus, it was classified as the nuclear type, whereas if β-catenin immunoreactivity was evident in the cytomembranes but not in the nucleus, the lesion was classified as the cytomembrane type.

### 4.4. Fluorescence In Situ Hybridization (FISH)

The sequence of the *Fn*-targeted probe, FUS664 (Cy3 labeled), was 5′-CTTGTAGTT CCGC(C/T)TACCTC-3′-(Chromosome Science Labo Inc., Sapporo, Japan). The 16 rRNA-targeted oligonucleotide probe was obtained from probe-Base (http://www.microbial-ecology.net/probebase/) [34,51]. The tissue sections were deparaffinized and then hybridized with the *Fn*-targeted probe at 46 ℃ for 2 h in accordance with the manufacturer’s protocol. After hybridization, the slides were washed in buffer (40% formamide, 0.9 M NaCl, 0.01% SDS, 20 mM Tris-HCl) for 10 min, then in phosphate-buffered saline, and counterstained with DAPI (Thermo Fisher Scientific, Tokyo, Japan).

SSA/P lesions were observed using a fluorescence microscope (DP72; Olympus, Tokyo, Japan; magnification ×200) and fluorescent signals were recorded throughout each lesion (at least five different visual fields). The number of signal dots for *Fn* was counted in each field and the average was calculated. When the average *Fn* signal count was greater than 3.0/field, the lesion was considered to be positive.

### 4.5. Stimulation for Caco2 Cells by Wnt Agonist SKL2001

Human intestinal epithelial cell line Caco2 was obtained from ATCC (Manassas, VA, USA) and cultured in RPMI 1640 medium (Invitrogen, Carlsbad, CA, USA) with 10% fetal bovine serum (Biowest, Nuaillé, France) in a humidified incubator at 37 °C with an atmosphere of 5% CO2. The cells were treated with Wnt agonist SKL2001 (40 µM, Selleck, Houston, USA) for 24 h.

Total RNA was isolated from the cells and reverse-transcribed using oligo-dT primer (Applied Biosystems, Branchburg, NJ). Thereafter, real-time RT-PCR was performed as previously reported [18]. In brief, the following sets of primers for human *REG Iα* and *glyceraldehydes-3-phosphate dehydrogenase* (*GAPDH*) were prepared: human REG Iα 5′-CTAGAGGCAACTGGAAAATACATGTCT-3′ (sense), 5′-GTTGGAGAGATGGTCCGGTTT-3′ (antisense), human GAPDH 5′-GAGTCAACGGATTTGGTCGT-3′ (sense), 5′-TTGATTTTGGAGGGATCTCG-3′ (antisense). The intensity of the fluorescent dye was determined, and the expression levels of *REG Iα* mRNA were normalized to those of *GAPDH* mRNA.

The treated Caco2 cell were also subjected to immunostaining of β-catenin. Details are mentioned in the Figure 4 legend. Briefly, the cells were fixed with methanol, incubated with anti-β-catenin antibody (dilution 1:100; Cell Signaling), followed by TRITC-labeled secondary anti-rabbit antibodies (dilution 1:1000; DAKO), and counterstained using Antifade Mountant with DAPI (Life Technologies, Carlsbad, CA, USA).

### 4.6. Statistical Analysis

All values were expressed as the mean ± SE. Significance of differences between two animal groups was analyzed by Mann–Whitney *U*-test. Correlations between two parameters were assessed by linear regression analysis. Differences were considered to be significant at *p* < 0.05.

## Figures and Tables

**Table 1:** Association between *Fn* presence and β-catenin, REG Iα, or Ki67 expression in SSA/P lesions.

	*Fusobacterium nucleatum*		*p* Value
	Negative	Positive	
**β-catenin expression**			0.001
Cytomembrane type	10	2	
Nuclear type	4	14	
**REG Iα expression score**	1.23 ± 0.15	1.85 ± 0.14	<0.01
**Ki67 labeling index**	7.0 ± 1.3	16.8 ± 2.3	<0.01

**Table 2:** Association between dysplastic change and β-catenin, REG Iα or Ki67 expression in SSA/P lesions.

	Dysplastic Change		*p* Value
	(−)	(+)	
**β-catenin expression**			0.025
Cytomembrane type	9	3	
Nuclear type	6	12	
**REG Iα expression score**	1.35 ± 0.16	1.85 ± 0.15	0.019
**Ki67 labeling index**	10.7 ± 2.5	15.1 ± 2.3	0.130

**Table 3:** Clinicopathological features of patients with SSA/Ps.

Gender		
	Male	16
	Female	14
**Age** (years, mean ± SE, range)		64.5 ± 2.2 (31–83)
**Tumor location**		
	Right side	26
	Left side	4
**Tumor size** (mm, mean ± SE, range)		21.1 ± 1.5 (6–40)
**Endoscopic morphology**		
	Is/Isp	2
	IIa	14
	LST-NG	14

LST-NG, laterally spreading tumor non-granular type.

## Data Availability

The data presented in this study are available upon reasonable request from the corresponding author via hfukui@hyo-med.ac.jp.
